# Characterization of two distinct early post-entry blocks to HIV-1 in common marmoset lymphocytes

**DOI:** 10.1038/srep37489

**Published:** 2016-11-23

**Authors:** Beatriz Pacheco, Luis Menéndez-Arias, Joseph Sodroski

**Affiliations:** 1 Centro de Biología Molecular Severo Ochoa, Consejo Superior de Investigaciones Científicas and Universidad Autónoma de Madrid, Madrid, Spain; 2 Department of Cancer Immunology and Virology, Dana-Farber Cancer Institute and Department of Microbiology and Immunobiology, Harvard Medical School, Boston, MA, USA; 3 Department of Immunology and Infectious Diseases, Harvard School of Public Health, Boston, MA, USA

## Abstract

In nature, primate lentiviruses infect humans and several Old World monkeys and apes. However, to date, lentiviruses infecting New World monkeys have not been described. We studied the susceptibility of common marmoset cells to HIV-1 infection and observed the presence of post-entry blocks to the early phase of HIV-1 infection in peripheral blood lymphocytes (PBLs) and a B lymphocytic cell line (B-LCL). The blocks present in these cells are dominant and phenotypically different from each other. In PBLs, the block occurs at the level of reverse transcription, reducing the accumulation of early and late transcripts, similar to the block imposed by TRIM5α. However, we have found that marmoset TRIM5α does not block HIV-1. In contrast, the restriction factor present in B-LCLs blocks HIV-1 replication at a later step, after nuclear entry, and inhibits integration. Additionally, we have identified an HIV-1 capsid mutant, N74D, that is able to escape the restriction in the marmoset B-LCLs. Our results suggest that the factors responsible for the blocks present in marmoset PBLs and B-LCLs are different. We propose the existence of at least two new restriction factors able to block HIV-1 infection in marmoset lymphocytes.

During evolution, host species have developed as part of their intrinsic immune system dominant-acting factors (known as restriction factors) that can block replication of viruses, including retroviruses. In response, the targeted viruses have evolved countermeasures to avoid restriction by these factors. This continuous host-versus-virus battle has led to the speciation of viruses.

The primate lentiviruses include the human immunodeficiency viruses type 1 and 2 (HIV-1 and HIV-2, respectively), and more than 40 different lentiviruses, collectively denominated simian immunodeficiency viruses (SIVs), that infect several species of Old World monkeys and apes of Africa. In nature, HIV-1 and HIV-2 infect humans, while HIV-1-related SIV_cpz_ and SIV_ora_ viruses infect chimpanzees and orangutans, respectively, and other SIV variants infect Old World monkeys^1^.

Some of the restriction factors that limit lentivirus host range have been described including TRIM5α, SAMHD1, MX2, BST2, and APOBEC3G (A3G)^2^,^3^,^4^,^5^,^6^,^7^,^8^,^9^,^10^. TRIM5α acts at an early post-entry step of the viral life cycle, recognizing the incoming viral capsid in a species-specific way and inducing accelerated uncoating of the capsid^9^,^11^. The main consequence of this recognition is a reduced accumulation of reverse transcription products that results in an abortive infection^9^,^12^. The cellular protein SAMHD1, a dendritic- and myeloid-cell-specific HIV-1 restriction factor, inhibits the viral life cycle at the reverse transcription step and is counteracted by the viral protein Vpx^3^,^5^. MX2 has been defined as a potent inhibitor of the early phase of HIV-1 infection^2^,^4^,^6^. The capsid region of the Gag protein dictates susceptibility to MX2, and the block to infection affects both nuclear accumulation and integration of the viral DNA. A3G is a member of the cytidine deaminase family that when incorporated into the virions can block replication at early post-entry steps of the viral cycle by various mechanisms^8^. By contrast, BST2 blocks replication at late steps in the viral cycle, inhibiting the release of the viral particles^7^,^10^. Several lines of evidence suggest the existence of additional restriction factors that block replication of lentiviruses.

New World monkeys are apparently resistant to infection by known lentiviruses. Some of the blocks to HIV-1 infection in common marmosets, a New World monkey, have been characterized. The earliest block to HIV-1 infection of common marmoset cells occurs at the level of virus entry, due to an inefficient recognition of common marmoset CD4 and CCR5 by the HIV-1 envelope glycoproteins^13^. Common marmoset A3G and BST2 also block HIV-1 replication^14^. Using a directed evolution method that takes advantage of the natural ability of the virus to mutate during replication, we have generated HIV-1 variants able to replicate in cells expressing common marmoset CD4 and CXCR4^15^, A3G or BST2^14^.

Here, we report the existence of two additional post-entry blocks in common marmoset primary lymphocytes and a B lymphocytic cell line (B-LCL). The blocks present in these cells are dominant and phenotypically different from each other. We identified an HIV-1 capsid mutant that is able to escape the block in the marmoset B-LCLs. We propose the existence of at least two restriction factors able to block HIV-1 infection in these cells. This work provides new insights into virus-host interactions. Understanding how these restriction factors operate and how the virus is able to escape from these blocks in different species could assist the development of novel interventions against HIV-1 infection.

## Results

### Post-entry block(s) to HIV-1 in common marmoset peripheral blood lymphocytes

**Figure 1:**
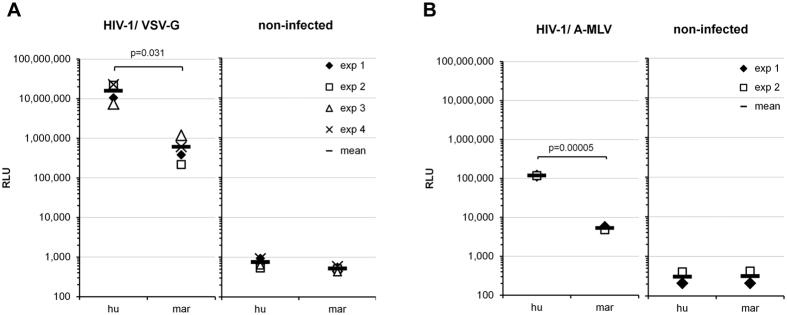
Post-entry block to HIV-1 in common marmoset PBLs. Infectivity of recombinant HIV-1 viruses expressing luciferase and pseudotyped with VSV-G (**A**) or A-MLV (**B**) envelope glycoproteins for PBLs from humans (hu) or common marmosets (mar). Forty-eight hours after the viruses were added to the PBLs, the infectivity of the viruses was determined by measuring the relative luciferase activity (RLU, relative luciferase units) in the cells. The charts show the results of four (**A**) or two (**B**) independent experiments and the means. Reported p-values (2-tailed) were obtained with an unpaired t-test.

Common marmosets are resistant to HIV-1 infection due to the presence of several barriers^13^,^14^. To investigate the presence of additional early blocks to HIV-1 infection in primary lymphocytes of common marmosets, we prepared single-cycle luciferase reporter viruses pseudotyped with the vesicular stomatitis virus G (VSV-G) envelope glycoprotein in 293T cells. These HIV-1 reporter viruses were used to challenge phytohemagglutinin (PHA-P)-activated peripheral blood lymphocytes (PBLs) from humans or common marmosets. As shown in Fig. 1A, in the marmoset PBLs, the infectivity of HIV-1 was more than 10-fold lower than in the human PBLs, suggesting the presence of a post-entry block in the marmoset PBLs.

The recombinant viruses used to test HIV-1 infectivity in PBLs were pseudotyped with VSV-G, which has been shown to mediate viral entry through clathrin-based endocytosis^16^,^17^,^18^. To evaluate whether the block to HIV-1 infection was envelope dependent, we prepared HIV-1 viruses pseudotyped with the amphotropic murine leukemia virus (A-MLV) envelope glycoprotein, which mediates viral entry through a mechanism that involves caveola-dependent endocytosis or macropinocytosis^19^,^20^. The infectivity of the viruses pseudotyped with the A-MLV envelope glycoprotein was lower in all the cells compared with VSV-G-pseudotyped viruses, probably due to the slower entry kinetics of the A-MLV envelope^19^. Nonetheless, with the A-MLV-pseudotyped viruses the strength of the blocks to HIV-1 infection in marmoset PBLs were similar to those observed with VSV-G-pseudotyped viruses (Fig. 1B). These results indicate that an early-phase block to HIV-1 infection exists in marmoset PBLs unrelated to the route of entry of the virus.

A block to HIV has been described in quiescent T cells^21^. One explanation of the low infectivity of HIV-1 in the marmoset PBLs is that the cells were not properly activated. However, we observed that with our activation protocol and under the cell culture conditions used in our assays, the marmoset PBLs proliferate efficiently, even better than the human PBLs. To quantitate the efficiency of proliferation of the human and marmoset PBLs we used a CellTrace^®^ Violet cell proliferation kit (Invitrogen). This dye readily diffuses into cells and binds cellular amines resulting in a stable, well-retained fluorescent label. With each cycle of proliferation the fluorescence signal in the cells will decrease. The decrease in fluorescence over time in the marmoset PBLs was more pronounced than in the human PBLs (Fig. S1). This result indicates that the marmoset PBLs proliferated more efficiently than the human PBLs, and argues against the possibility that the observed block was due to insufficient activation of the marmoset PBLs.

### Post-entry block to HIV-1 in marmoset B lymphocytic cell lines

**Figure 2:**
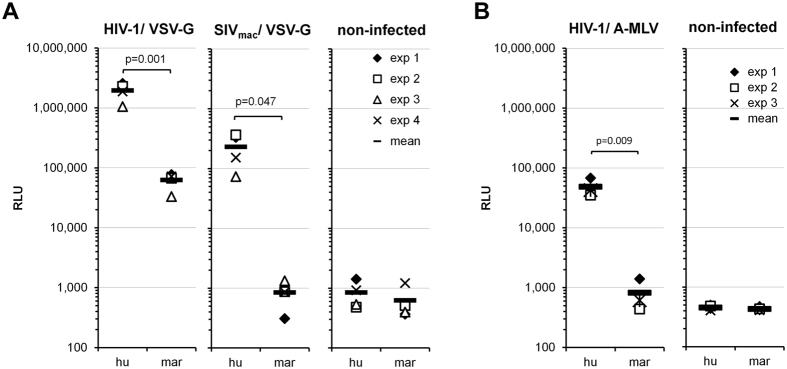
Post-entry block to HIV-1 in common marmoset B-LCLs. Human (hu) or marmoset (mar) B-LCLs were challenged with HIV-1 or SIV_mac_ luciferase reporter viruses pseudotyped with VSV-G (**A**) or the A-MLV envelope glycoprotein (**B**). Forty-eight hours later, the infectivity of the viruses was determined by measuring the relative luciferase activity (RLU) in the B-LCLs. The charts show the results of four (**A**) or three (**B**) independent experiments. Reported p-values (2-tailed) were obtained with an unpaired t-test.

We tested a marmoset B lymphocytic cell line (B-LCL) to evaluate whether a block to HIV-1 infection similar to that found in marmoset PBLs is present in this cell line. Human and marmoset B-LCLs were challenged with single-cycle HIV-1 luciferase reporter viruses pseudotyped with VSV-G. Marmoset B-LCLs exhibited a greater than 10-fold reduction in the infectivity of HIV-1 compared to that in human B-LCLs (Fig. 2A), reminiscent of the results obtained in PBLs. We also found that the infectivity of SIV_mac_ was reduced more than 100-fold in the marmoset B-LCLs compared to that in human B-LCLs (Fig. 2A). Similarly, the infectivity of HIV-1 viruses pseudotyped with the A-MLV envelope glycoprotein was also more than 10-fold lower in the marmoset B-LCLs than in the human B-LCLs (Fig. 2B). These results indicate the existence of a specific block to HIV-1 as well as to SIV_mac_ in the marmoset B-LCLs.

### The post-entry blocks to HIV-1 in marmoset PBLs and B-LCLs are dominant

**Figure 3:**
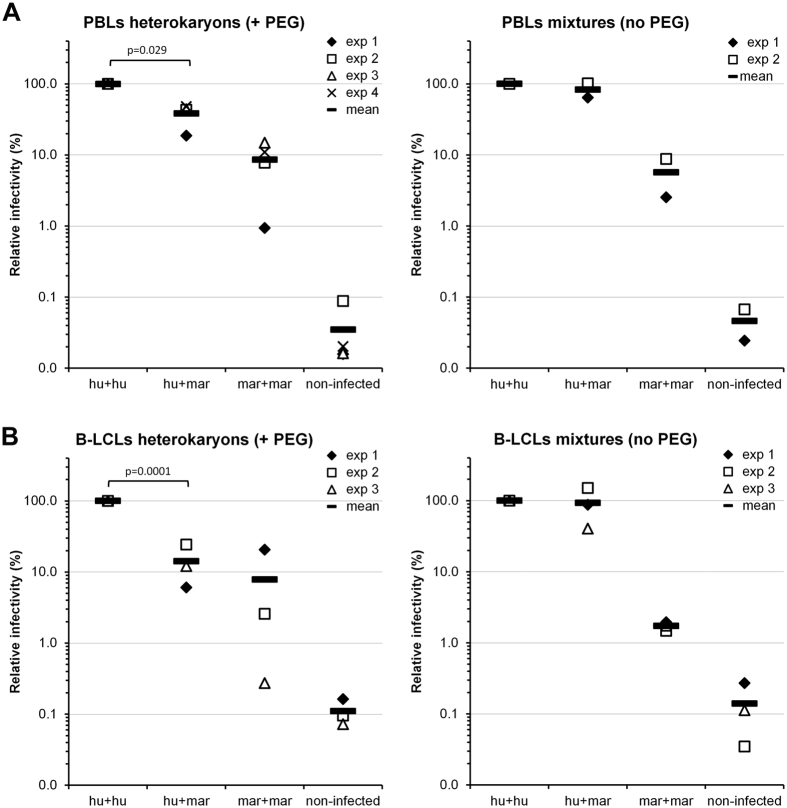
The post-entry block(s) to HIV-1 in marmoset PBLs and B-LCLs are dominant. (**A**) Heterokaryons or homokaryons of human (hu) and marmoset (mar) PBLs were produced by treatment with PEG. As a control, mixtures of human and marmoset cells were treated the same way but without adding PEG. The fused and non-fused cell mixtures were challenged with HIV-1 luciferase reporter viruses pseudotyped with VSV-G. Forty-eight hours later, the infectivity of the viruses was determined by measuring the relative luciferase activity in the cells. The infectivity in the human-human homokaryons is normalized to 100%, and the relative infectivity in the other homo-heterokaryons is shown. The results of four (+PEG) and two (no PEG) independent experiments and their means are shown. (**B**) Human and marmoset B-LCLs were fused with PEG to generate hetero- or homokaryons. As a control, mixtures of human and marmoset cells were treated the same way but without adding PEG. The fused and non-fused cell mixtures were challenged with HIV-1 luciferase reporter viruses pseudotyped with VSV-G. Forty-eight hours later, the infectivity of the viruses was determined by measuring the relative luciferase activity in the cells. The infectivity in the human-human homokaryons is normalized to 100%, and the relative infectivity in the other homo-heterokaryons is shown. The results of three independent experiments and their means are shown. Reported p-values (2-tailed) were obtained with an unpaired t-test.

To determine whether the block to HIV-1 infection in marmoset PBLs was due to the presence of a dominant factor and not to a deficiency in a factor needed for HIV-1 replication, we prepared heterokaryons and homokaryons of restricted and unrestricted PBLs using polyethylene glycol (PEG). As a control, we also prepared mixtures of human and marmoset PBLs that were not treated with PEG in parallel, and tested the infectivity of HIV-1 in the PEG-fused and non-fused cell mixtures. If the block were due to the presence of a dominant factor, we would expect to observe low HIV-1 infectivity in heterokaryons formed between restricted (marmoset) and unrestricted (human) cells. On the contrary, if the block were due to the absence of a factor needed for HIV-1 infection, we would expect to see HIV-1 infectivity at levels close to those seen in unrestricted cells or in the non-fused human-marmoset cell mixtures. As shown in Fig. 3A, HIV-1 infectivity was reduced in heterokaryons formed between human and marmoset PBLs by 2.6-fold compared to human-human homokaryons. We did not observe a significant reduction in infectivity in the non-fused human-marmoset cell mixtures (Fig. 3A, right). These results suggest that the early block to HIV-1 present in marmoset PBLs is most likely due to the presence of a dominant-acting factor, as is the case of the block present in rhesus cells^22^. The block in human-marmoset heterokaryons was not as pronounced as in marmoset-marmoset homokaryons, which showed a 12-fold lower infectivity compared to human-human homokaryons. This effect can be attributed to the formation of some human-human homokaryons and/or to incomplete cell-cell fusion. We have not been able to assess the efficiency of heterokaryon formation in our infection experiments because treatment of the cells with dyes that would allow this measurement resulted in background levels of infectivity in the PEG-treated cells. However, in pilot experiments using Vibrant DiO and Dil cell labeling solutions (Molecular Probes) to evaluate cell-cell fusion, the efficiency of heterokaryon formation ranged from 60% to 80%. Thus, it is likely that most cells have fused under the conditions of our experiment.

To evaluate whether the block present in the marmoset B-LCLs was also caused by a dominant factor, we prepared heterokaryons and homokaryons of marmoset and human B-LCLs. As a control, we also prepared mixtures of human and marmoset B-LCLs that were not treated with PEG in parallel. We challenged the PEG-fused cells and the non-fused mixtures with HIV-1 luciferase reporter viruses pseudotyped with VSV-G. The human-marmoset heterokayons (PEG-treated cells) exhibited a reduced permissivity (7-fold decrease) to HIV-1 compared with the human-human homokaryons (Fig. 3B, left). However, we didn’t observe a substantial reduction in HIV-1 infectivity in the human-marmoset B-LCLs mixtures that had not been treated with PEG (Fig. 3B, right). These results suggest that the block to HIV-1 infection in the marmoset B-LCLs is due to a dominant factor, not to the lack of a factor needed for HIV-1 infection.

### The post-entry block to HIV-1 in marmoset PBLs reduces accumulation of early and late reverse transcription products

**Figure 4:**
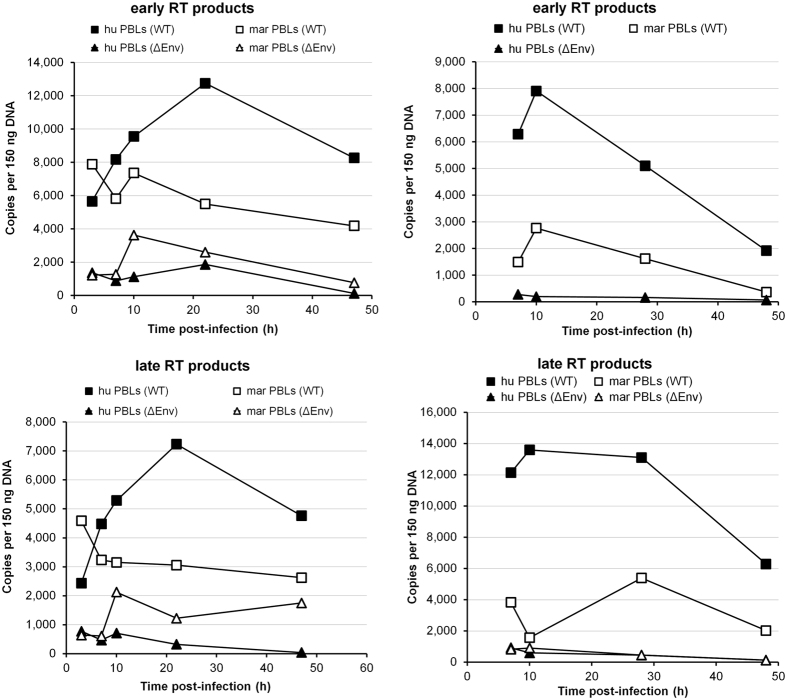
The post-entry block to HIV-1 in marmoset PBLs reduces the accumulation of early and late reverse transcription products. Human (hu) or marmoset (mar) PBLs were challenged with single-cycle HIV-1 viruses pseudotyped with VSV-G pretreated with DpnI for 1 h. As a control, cells were also challenged with viruses lacking an envelope glycoprotein (ΔEnv). Two hours post-infection, the cells were thoroughly washed 3 times with PBS to remove excess virus. At different time points after infection, cells were collected and total DNA isolated. The amounts of early and late RT products were quantified by real-time qPCR using TaqMan^®^ chemistry. The results of two independent experiments are shown.

When complemented in *trans* with VSV-G, the single-cycle reporter viruses used in our experiments are able to enter the target cells. If the cells are permissive to infection, the virus will be able to complete all the early events in the replication cycle, including reverse transcription, nuclear entry, integration and reporter gene expression, but will not produce new viral particles. To define the step of the early HIV-1 replication cycle that is blocked in marmoset PBLs, we challenged human or marmoset PBLs with single-cycle HIV-1 green fluorescent protein (GFP) reporter viruses pseudotyped with VSV-G and analyzed the formation of early and late reverse transcription (RT) products by real-time qPCR at different times post-infection. As a control, we also infected human and marmoset PBLs with viruses lacking an envelope glycoprotein (ΔEnv). As expected, in human PBLs we detected an increase of early and late RT products over time (Fig. 4). On the contrary, in marmoset PBLs, the amounts of early and late RT products exhibited slight decreases over time (Fig. 4). These observations suggest that the factor responsible for the HIV-1 block in marmoset PBLs acts at an early step after virus entry, before or during reverse transcription. This factor may directly or indirectly disrupt the reverse transcription process itself or degrade the products of reverse transcription.

### TRIM5α and TRIMCyp are not responsible for the early block to HIV-1 in marmoset PBLs

**Figure 5:**
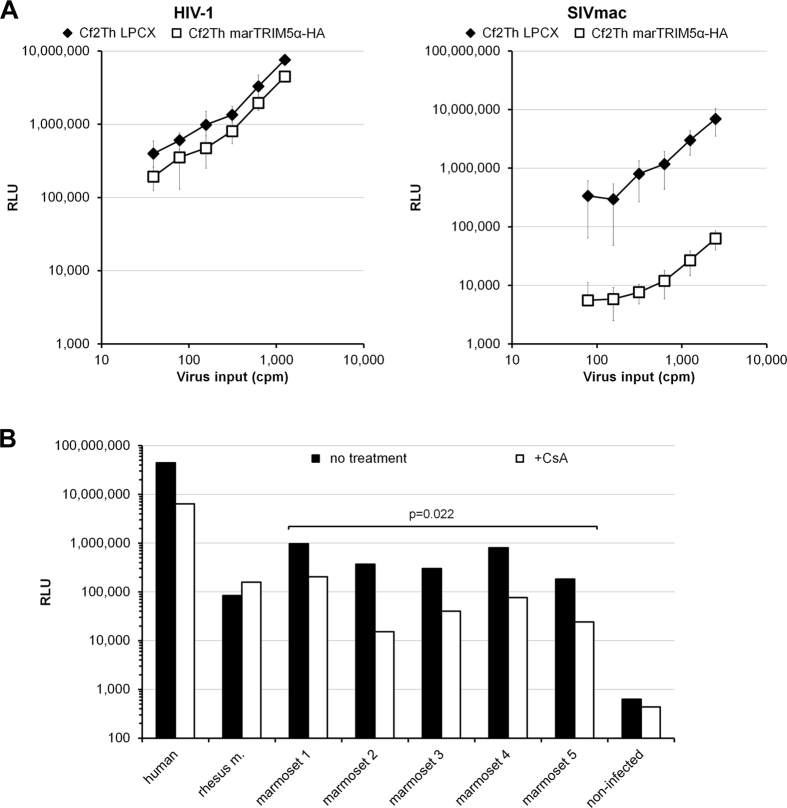
Effect of TRIM5α and cyclosporine A on the infectivity of HIV-1. (**A**) Cf2Th cell lines stably expressing marmoset TRIM5α with an HA tag, were challenged with single-cycle HIV-1 (left) or SIV_mac_ (right) luciferase reporter viruses. Forty-eight hours post-challenge, the infectivity of the HIV-1 viruses was determined by measuring the relative luciferase activity (RLU) in the cells. Data shown are the means ± standard deviations of three independent experiments. (**B**) Human, rhesus macaque or common marmoset PBLs were challenged with single-cycle HIV-1 viruses pseudotyped with VSV-G in the presence or absence of 5 μM cyclosporine A (CsA). PBLs from five different common marmoset donors were tested. Forty-eight hours post-challenge, the infectivity of the HIV-1 viruses was determined by measuring the relative luciferase activity (RLU) in the cells. Reported p-value (2-tailed) for the 5 common marmoset PBLs (untreated versus CsA treated) were obtained with a paired t-test.

The early post-entry block to HIV-1 infection in marmoset PBLs resembles the early block imposed by TRIM5α and TRIMCyp in some species^9^,^23^. To test whether TRIM5α from the common marmoset accounted for the observed block to HIV-1, we cloned marmoset TRIM5α (marTRIM5α) and prepared stable cell lines expressing this protein. The expression of the HA-tagged marmoset TRIM5α in Cf2Th cells was documented by Western blot (data not shown). The infectivity of HIV-1 and SIV_mac_ in these stable cell lines was tested with single-cycle luciferase reporter viruses. We observed only minimal differences (about a 1.7-fold decrease) in the infectivity of HIV-1 in cells expressing marTRIM5α compared to control cells transduced with the empty vector (Fig. 5A, left), suggesting that marTRIM5α does not efficiently block HIV-1. However, we observed that marTRIM5α was able to strongly block SIV_mac_ infection (Fig. 5A, right), which confirms that marTRIM5α is active in these cell lines.

The *TRIMCypA* gene is the result of a retrotransposition of a *CypA* gene into the *TRIM5* gene in some monkey species. The encoded protein TRIMCyp has been shown to have antiviral activity against some retroviruses^24^,^25^,^26^,^27^,^28^. A *CypA* retrogene has not been found proximal to or downstream of *TRIM5* in marmosets^29^ and our attempts to amplify a TRIMCypA cDNA from common marmoset mRNA were unsuccessful. To rule out TRIMCypA or a CypA-containing protein as a potential factor responsible for the early post-entry block in marmoset PBLs, we carried out the infection of PBLs from five common marmosets in the presence or absence of cyclosporine A (CsA), a drug that binds CypA and inhibits its binding to the HIV-1 capsid. As shown in Fig. 5B, treatment with CsA reduced the infectivity of HIV-1 in human PBLs, as previously reported^30^. Similarly, treatment with CsA significantly reduced the infectivity of HIV-1 in marmoset PBLs, suggesting that the early block in marmoset PBLs was not due to the action of TRIMCyp or other similar protein containing CypA. On the contrary, our results suggest that CypA contributes to the infectivity of HIV-1 in human and marmoset PBLs. In contrast, in rhesus macaque PBLs, addition of CsA slightly increased the infectivity of HIV-1, an effect that could be due to the presence of TRIMCyp in this rhesus macaque^24^,^25^,^27^,^28^ or may reflect the contribution of CypA to TRIM5α-mediated restriction.

### The post-entry block to HIV-1 in marmoset B-LCLs acts at a step after reverse transcription

**Figure 6:**
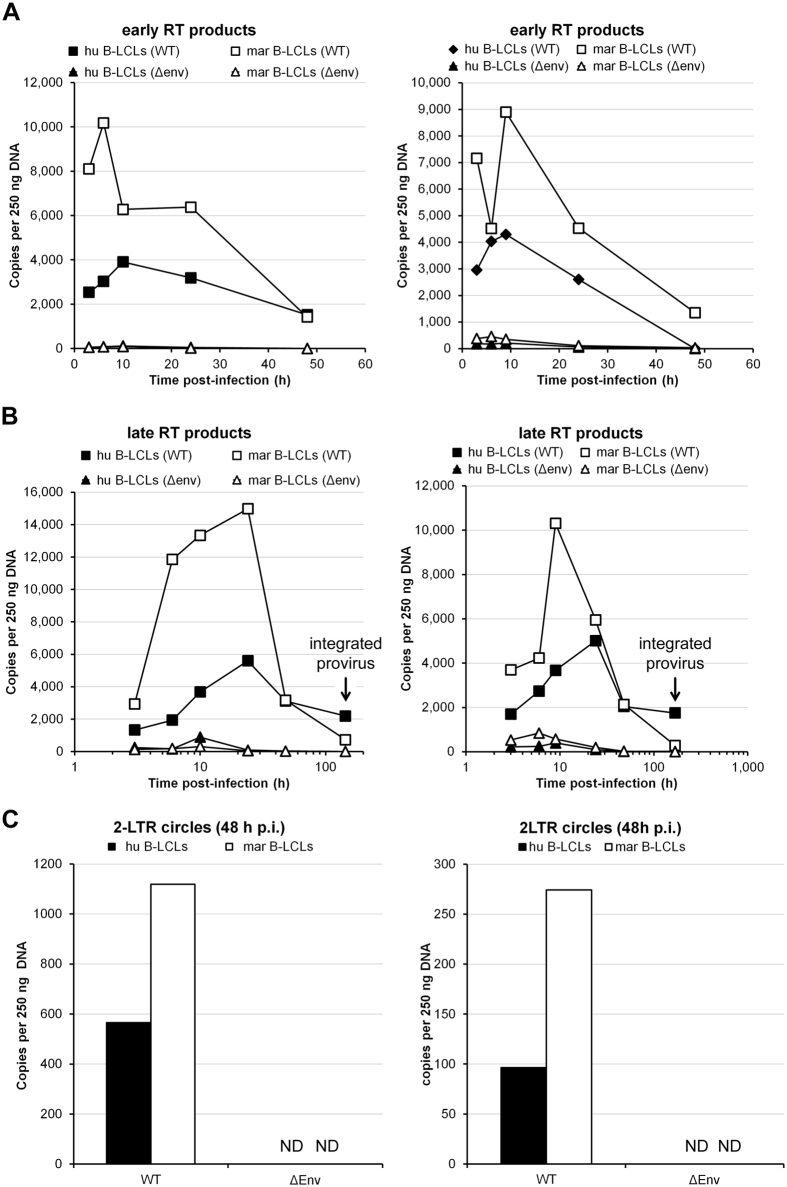
HIV-1 is blocked in marmoset B-LCLs after reverse transcription. Human (hu) or marmoset (mar) B-LCLs were challenged with single-cycle HIV-1 GFP reporter viruses pseudotyped with VSV-G (indicated as WT), or as a control with viruses lacking an envelope glycoprotein (ΔEnv). Viruses were pretreated with DNase I for 1 h. Two hours post-infection (p.i.), the cells were thoroughly washed 3 times with PBS. At different time points after infection, cells were collected and total DNA isolated. The amounts of early (**A**) and late (**B**) RT products and 2-LTR circles (**C**) were quantified by real-time qPCR using TaqMan^®^ chemistry. ND, not detectable. The results of two independent experiments are shown.

Our results indicated that a post-entry block to HIV-1 infection exists in marmoset B-LCLs. To evaluate whether this block resembles the one observed in the marmoset PBLs, we analyzed by real-time qPCR the formation of early and late RT products at different time points after the infection of B-LCLs with HIV-1 single-cycle GFP reporter viruses. Surprisingly, the results in the B-LCLs did not recapitulate the results obtained in marmoset PBLs. In marmoset B-LCLs, the formation of early and late RT products was considerably higher than in human B-LCLs (Fig. 6A and B), in contrast with the data obtained in the PBLs (Fig. 4). These results indicated that the factor responsible for the block to HIV-1 in the marmoset B-LCLs was acting after reverse transcription. We attempted to measure integration of the proviral DNA with the Alu-PCR assay^31^. However, we were unable to amplify any viral DNA in the infected human or marmoset B-LCLs with this test. As a surrogate of Alu PCR to measure the integration of viral DNA, we quantified the amount of proviral DNA six or seven days after infection, using the same primers as those used for the late RT assay. At this time point, all the viral DNA remaining in the cells should be integrated in the genome^32^. Although at early time points the amount of viral DNA was higher in the marmoset than in the human cells, after 24 hours in culture we observed a substantial drop in the amount of viral DNA in the marmoset B-LCLs; at six/seven days post-infection, the amount of viral DNA in the marmoset B-LCLs was lower than in the human B-LCLs (Fig. 6B). These observations suggest that the block to HIV-1 in the marmoset B-LCLs occurs before or during integration.

2-LTR circles are a dead-end product formed only in the nucleus, so their presence is frequently used as an indicator of nuclear import. At 48 hours post-infection, the amount of 2-LTR circles in the marmoset B-LCLs was higher than in the human B-LCLs (Fig. 6C). These data suggest that the nuclear import of the preintegration complex was not blocked in the marmoset B-LCLs, consistent with the hypothesis that the HIV-1 block in B-LCLs was at the level of integration.

### Some HIV-1 capsid mutants escape the post-entry block in marmoset B-LCLs

**Figure 7:**
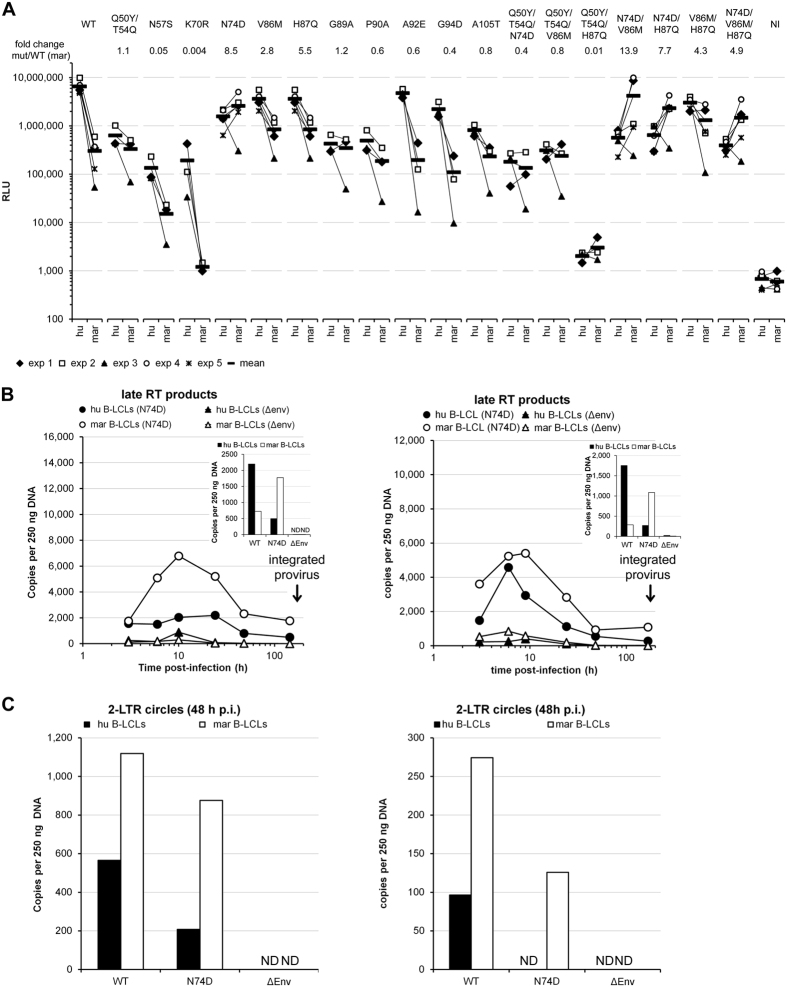
Infectivity of HIV-1 capsid mutants in B-LCLs. (**A**) Human (hu) or marmoset (mar) B-LCLs were challenged with single-cycle HIV-1 luciferase reporter viruses pseudotyped with VSV-G and containing the indicated changes in their capsids. Forty-eight hours later, the infectivity of the viruses was determined by measuring the relative luciferase activity (RLU) in the cells. The results of three or five independent experiments and their means are shown. The mean fold change in infectivity for each mutant relative to WT virus in the marmoset B-LCLs is shown above the chart. NI, non-infected. (**B**,**C**) Human (hu) or marmoset (mar) B-LCLs were challenged with the single-cycle HIV-1 N74D capsid mutant as in the experiments shown in Fig. 6A,B and C. At different time points after infection, cells were collected and total DNA isolated. The amounts of late RT products (**B**) and 2-LTR circles (**C**) were quantified by real-time qPCR using TaqMan^®^ chemistry. The results of two independent experiments are shown. The inserts in panel B show the amount of viral cDNA six (left) or seven (right) days post-infection (integrated proviruses) for the WT and N74D viruses. ND, not detectable.

Some restriction factors targeting early events in the replication cycle, like TRIM5α and MX2, are known to specifically bind the viral capsid. We prepared a panel of HIV-1 capsid mutants that have been described previously to affect early events following virus entry^33^,^34^,^35^,^36^,^37^,^38^,^39^. We tested the ability of the capsid mutants to infect human and marmoset B-LCLs. The infectivity of the WT viruses in the marmoset B-LCLs was more than 10 times lower than in the human B-LCLs (Fig. 7A), in agreement with the results described above. Of interest, we observed that the capsid mutants containing the N74D change, either alone or in combination with the V86M and/or H87Q capsid changes, exhibited increased infectivity (4.9- to 13.9-fold increase) in the marmoset B-LCLs compared to WT viruses (Fig. 7A). However, the combination of the N74D change with the Q50Y and T54Q changes was detrimental to the infectivity of the virus. Apparently, the N74D change in the capsid allows HIV-1 to escape the action of the factor responsible for the early post-entry block in the marmoset B cell lines. The V86M and H87Q capsid mutants also exhibited increased infectivity in the marmoset cells compared to WT viruses, although the escape was not as efficient as that of the N74D mutant.

To examine the mechanism of escape of the N74D capsid mutant in more detail, we studied the kinetics of the synthesis of reverse transcription products by real-time qPCR. The production by the N74D mutant of late reverse transcripts was higher in the marmoset B-LCLs compared to the human B-LCLs (Fig. 7B), as was the case for the WT virus (Fig. 6B). At early times post-infection, the N74D mutant showed a reduced capacity to synthesize late RT products compared to the WT virus in the marmoset B-LCLs (Figs 6B and 7B). However, at late time points after the infection (6/7 days), when all the remaining viral DNA should be integrated, the amount of proviral DNA in the marmoset cells was higher for the N74D mutant than for the WT virus (Fig. 7B, inset). These results agree with our observation that the marmoset B-LCLs were more permissive to the N74D mutant than to the WT HIV-1 virus. The N74D mutant exhibited reduced nuclear import (formation of 2-LTR circles) in human and marmoset B-LCLs (Fig. 7C), suggesting that the N74D mutant exhibited an advantage over the WT virus around the time of integration.

### Characterization of the post-entry block to HIV-1 in marmoset B-LCLs

**Figure 8:**
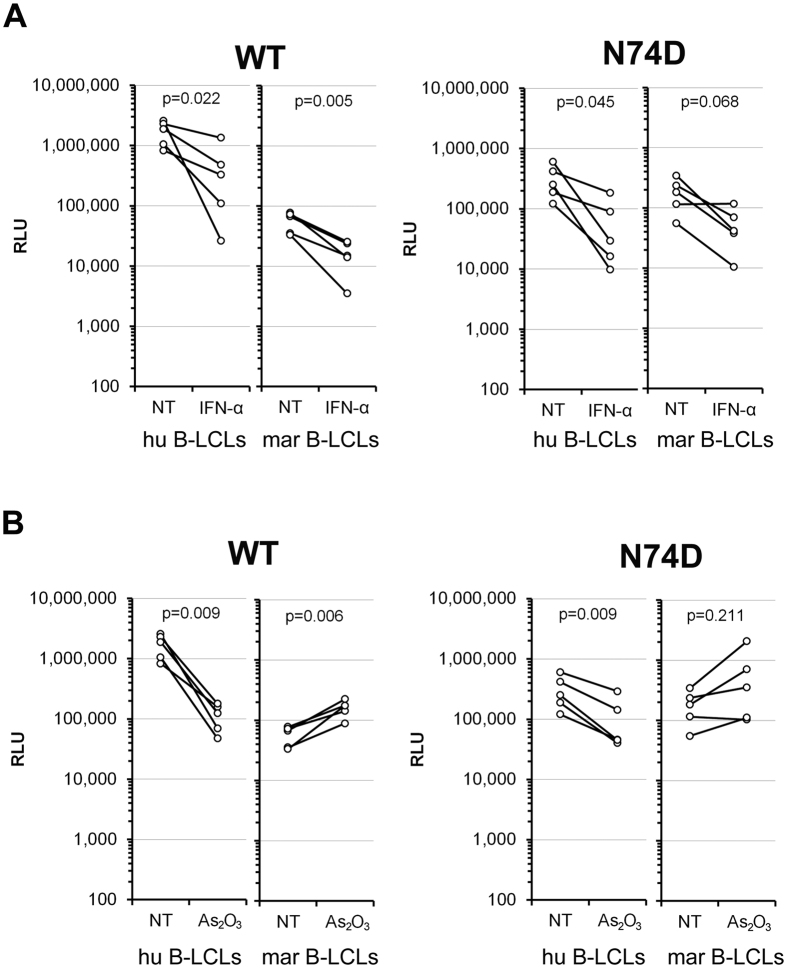
Characterization of the post-entry block to HIV-1 in B-LCLs. Human (hu) or marmoset (mar) B-LCLs were challenged with single-cycle HIV-1 WT or N74D capsid mutant luciferase reporter viruses pseudotyped with VSV-G. (**A**) Cells were pre-treated with 500 U/ml of universal IFN-α for 24 hours before the infection. (**B**) Cells were pre-treated with 4 μM As_2_O_3_ for 3 hours before the infection. The cells were maintained in the presence of the treatment for 24 hours after the viral challenge. Forty-eight hours post-challenge, the infectivity of the viruses was determined by measuring the relative luciferase activity (RLU) in the cells. The results of five independent experiments are shown. Reported p-values (2-tailed) were obtained with a paired t-test (untreated versus treated cells). NT, no treatment.

To assess if interferon-α (IFN-α) plays a role in the post-entry block to HIV-1 in marmoset B-LCLs, we pretreated the cells with 500 U/ml of universal IFN-α for 24 h and then challenged them with VSV-G-pseudotyped HIV-1 luciferase reporter viruses. Treatment of human and marmoset B-LCLs with IFN-α significantly decreased the infectivity of WT HIV-1 (Fig. 8A). IFN-α treatment also decreased the infectivity of the escape mutant N74D (Fig. 8A). Apparently, IFN-α treatment results in a general decrease in HIV-1 infectivity in these B-LCLs. However, the decrease of infectivity observed in the marmoset B-LCLs for the N74D mutant was statistically less significant (p = 0.068), suggesting that in the marmoset cells this mutant was affected to a lesser degree by IFN-α treatment.

Previous studies have shown that treatment with As_2_O_3_ rescues retrovirus infectivity in cells expressing TRIM5α^40^. This treatment has also been shown to suppress the antiviral activity of Lv4 and a post-entry inhibitory factor present in dendritic cells^41^,^42^. We analyzed the effect of As_2_O_3_ treatment on the infectivity of HIV-1 in the B-LCLs. The cells were preincubated with 4 μM As_2_O_3_ for 3 hours and then challenged with VSV-G-pseudotyped HIV-1 luciferase reporter viruses. In the human B-LCLs, treatment with As_2_O_3_ significantly decreased the infectivity of WT HIV-1 and the N74D mutant (Fig. 8B) by more than 10-fold and about 3-fold, respectively. On the contrary, in the marmoset B-LCLs, As_2_O_3_ treatment slightly increased the infectivity of HIV-1 WT as well as N74D mutant (Fig. 8B). Thus, in the presence of As_2_O_3_, HIV-1 infectivity is at least as great in the marmoset B-LCLs as in the human B-LCLs.

## Discussion

Here we identify and characterize two post-entry blocks operative against HIV-1 that are present in common marmoset lymphocytes. These blocks appear to be effective regardless of the entry pathway used by the virus (Figs 1 and 2). Infections of heterokaryons (Fig. 3) suggest that both blocks are attributable to the presence of dominant-acting factor(s) that inhibit HIV-1 replication in these cells.

To date, a few dominant blocks to the early phase of lentivirus infection have been described in cells of different species: Lv1 (later identified as TRIM5α), Lv2, Lv3, Lv4, SAMHD1 and MX2^2^,^3^,^4^,^5^,^6^,^9^,^12^,^22^,^42^,^43^,^44^. The identity of the factors responsible for the Lv2, Lv3 and Lv4 activities are currently unknown. TRIM5α acts at an early post-entry step in the viral life cycle, inducing premature uncoating of the capsid and reducing the formation of early and late reverse transcription products^9^,^11^. The first barrier to HIV-1 that we describe here is present in primary lymphocytes isolated from the peripheral blood of common marmosets and, like TRIM5α and TRIMCyp, disrupts the formation of viral reverse transcripts. However, our data failed to implicate either TRIM5α or TRIMCyp in this block in common marmoset cells (Fig. 5A and B). This block is also phenotypically distinct from those instituted by other previously described dominant-acting factors, such as MX2, Lv2, Lv3 or Lv4. SAMHD1 has also been reported to block lentiviruses at an early post-entry step, blocking reverse transcription by depleting the dNTP pool in cells of the myeloid lineage and quiescent CD4+ T lymphocytes^3^,^5^,^45^,^46^. However, SAMHD1 has been shown to be inactive due to phosphorylation in activated T lymphocytes^47^,^48^; as our marmoset PBLs are activated, SAMHD1 seems an unlikely candidate for the observed block. Together, our results suggest the presence of a novel dominant-acting restriction factor in common marmoset PBLs. Following the tradition for naming dominant-acting factors with anti-lentiviral activity of unknown identity, we suggest the name Lv5.

In marmoset B-LCLs, we detected the presence of another early phase block to HIV-1. In contrast to the block observed in marmoset PBLs, the synthesis of HIV-1 reverse transcription products is an efficient process in the marmoset B-LCLs, even more so than in human B-LCLs (Fig. 6). The formation of 2-LTR circles also occurs normally in these cell lines, indicating that the factor responsible for this second block likely acts after nuclear import and before or during integration of the viral DNA (Fig. 6). Thus, the factor responsible for the marmoset B-LCL block probably exerts its action in the nucleus. The mechanism of action of this factor resembles the recently reported Lv4 activity that blocks viruses of the SIV_mac_/SIV_sm_/HIV-2 lineage in human blood cells^42^. In that work, Pizzato and colleagues also showed that the Lv4 activity targets the capsid of SIV_mac_, SIV_sm_, and HIV-2. In our study, we found that the N74D and, to a lesser degree, the V86M and H87Q capsid mutants escape the block present in marmoset B-LCLs (Fig. 7A). This observation suggests that the factor responsible for this anti-HIV-1 activity might either directly target the capsid or be influenced by a capsid-specified process. Lentiviruses like HIV-1 are able to infect non-dividing cells and have evolved strategies to enter the nucleus through the nuclear pore complex (NPC). The molecular mechanism by which HIV-1 pre-integration complexes (PICs) traverse the NPC is under active investigation. The role of CA in nuclear import has been highlighted by several studies. Although for reverse transcription to occur, a partial uncoating of the viral capsid is necessary, different studies suggest that significant amounts of CA remain in the preintegration complex (PIC) and are probably translocated into the nucleus^49^,^50^,^51^,^52^. HIV-1 has been shown to mainly interact with NUP358/RANBP2, NUP153 and TNPO3 to enter the nucleus^53^,^54^. However, the N74D mutant seems to be less sensitive to TNPO3 and Nup358/RANBP2 depletion and instead uses different nucleoporins like NUP155 to enter the nucleus^32^. A truncated form of CPSF6 that is expressed in the cytoplasm has been identified as a potent inhibitor of HIV-1 that blocks nuclear import of the PIC^32^. HIV-1 harboring the N74D capsid change fails to interact with CPSF6 and evades this nuclear import restriction^32^. It is unlikely that marmoset CPSF6 factor is responsible for the post-entry block in marmoset B-LCLs, as nuclear import appears to be unaffected. In addition, there is 100% identity between marmoset and human CPSF6. The N74D mutant has been shown to enter the nucleus though an altered nuclear entry pathway that results in integration in genomic regions that are sparse in transcription units compared to WT viruses that integrate in gene-rich chromosomal regions^55^,^56^. Thus, the use of a different nuclear entry pathway by the N74D mutant might contribute to the escape from the Lv4-like activity in the marmoset B-LCLs. One model is that the factor responsible for the Lv4 anti-viral activity might be associated with gene-rich regions of the chromosomes.

Arsenic trioxide treatment has been shown to suppress the antiviral activity of TRIM5α, Lv4, and a post-entry inhibitory factor present in dendritic cells (DC)^40^,^41^,^42^. We found that treatment with arsenic specifically increased the infectivity of HIV-1 in the marmoset B-LCLs, whereas HIV-1 infectivity decreased in the human B-LCLs; the observed decrease in the human B-LCLs may have possibly resulted from non-specific toxic effects of the treatment. The mechanism by which arsenic trioxide relieves the restriction by TRIM5α, Lv4, or the DC factor is unknown. Arsenic could be targeting a common cofactor needed for all three restrictions, or it may be acting via a different mechanism in each case. Arsenic has been implicated in several cellular pathways like DNA repair inhibition, cell cycle disruption and ubiquitin dysregulation^57^. Trivalent arsenic has been shown to selectively interact with DNA repair proteins containing zinc finger motifs with at least 3 cysteine residues^58^. Such proteins would be expected to reside in the nucleus and could be candidates for the restriction factor in marmoset B-LCLs.

In summary, we have demonstrated the existence of two post-entry blocks in marmoset lymphocytes. The first block (Lv5) is found in primary lymphocytes and acts at the level of reverse transcription. The second (Lv4-like) is present in a B cell line and blocks HIV-1 infection at the level of viral DNA integration. Our data leave open the possibility that the Lv4-like block is also present in primary marmoset lymphocytes. Identification of the factors responsible for the Lv4-like and Lv5 activity and elucidation of their molecular mechanism of action could assist understanding of the early phase of HIV-1 infection and the development of interventions.

## Materials and Methods

### Cell lines and reagents

293T cells were obtained from the American Type Culture Collection and maintained in DMEM supplemented with 10% fetal bovine serum (DMEM-10). The human and common marmoset B-LCLs were obtained from the Coriell Institute for Medical Research (cat# GM00607 and GM07404, respectively) and maintained in RPMI1640 supplemented with 10% fetal bovine serum (RPMI-10).

### Peripheral blood lymphocytes

Peripheral blood lymphocytes (PBLs) were isolated from whole blood samples using Ficoll-Paque Plus gradients according to the manufacturer’s instructions. After isolation, the cells were activated for 3 days with 1 μg/ml of phytohemagglutinin P and cultured in complete lymphocyte medium (RPMI1640 supplemented with 20% fetal bovine serum, 10% IL-2 (Hemagen), 25 mM HEPES, 2 mM glutamine and antibiotics). The adherent cells were discarded. As the amount of blood that we can obtain from marmosets is limited, the activated PBLs (human, marmoset and rhesus) were generally grown for a few days in complete lymphocyte medium to obtain sufficient cells for the infection experiments.

Human fresh whole blood samples were obtained from healthy anonymous volunteers through Research Blood Components, LLC (Boston, MA, USA) or the Transfusion Center of the Community of Madrid (Spain). Proper informed consent was obtained from each donor according to local regulations.

Common marmoset and rhesus macaque blood samples were obtained from the New England Primate Research Center or the German Primate Center. For some experiments, frozen marmoset peripheral blood mononuclear cells were obtained from the Netherlands Biomedical Primate Research Center.

### Viruses

HIV-1 and SIV_mac_ single-cycle luciferase or GFP reporter viruses were prepared by transfection of 293T cells by the calcium phosphate method. The plasmids used for preparation of the viruses were: the pCMVΔP1ΔenvpA packaging plasmid, a Rev-expressing plasmid and pHIvec2.luc or pHIvec2.GFP vectors for HIV-1^59^,^60^, or pSIV1.GFP or pSIvec1.luc plus a Rev-expressing plasmid for SIVmac^59^. All viruses were pseudotyped with the VSV-G envelope glycoprotein by cotransfecting with a pHCMV plasmid expressing VSV-G.

Forty-eight hours after transfection, supernatants containing reporter viruses were harvested and cleared by low-speed centrifugation. The amount of virus in the supernatants was quantified by measuring reverse transcriptase activity.

### Single-cycle infectivity assay

The efficiency of a single cycle of HIV-1 or SIV_mac_ infection was measured by using recombinant reporter viruses expressing firefly luciferase and pseudotyped with VSV-G or A-MLV envelope glycoproteins.

Cells were seeded at a density of 10^5^ (for the B-LCLs) or 5 × 10^5^ (for the PBLs) cells/well in 96-well luminometer-compatible tissue culture plates. The cells were challenged with 10,000 (for the B-LCLs) or 50,000 (for the PBLs) reverse transcriptase units (cpm) of viruses in a total volume of 100 μl. Forty-eight hours later, the medium was removed and cells were lysed with 30 μl of passive lysis buffer (Promega). Luciferase activity was measured using an EG&G Berthold LB 96 V microplate luminometer or a BMG Labtech FLUOstar Optima microplate reader.

### Heterokaryon assay

Equal amounts of human or marmoset PBLs or B-LCLs were mixed and fused by treatment with polyethylene glycol 1500 (PEG1500) (Roche) according to the manufacturer’s instructions. Control cell mixtures were treated according to the same protocol except that buffer (75 mM Hepes pH 7.5) was substituted for PEG1500. After fusion, the cells were resuspended in medium and plated in 96-well plates compatible with luminescence. After about 3 hours of recovery, the cells were challenged with HIV-1 luciferase reporter viruses pseudotyped with VSV-G. The level of infectivity was determined forty-eight hours later by measuring the luciferase activity in the cells.

### Analysis of reverse transcription products by real-time qPCR

The viral stocks used for the infections were pretreated with 20 U/ml of DpnI (New England Biolabs) or 10 U/ml of DNase I (Promega) for 1 h at 37 °C to eliminate plasmid DNA that might be present in the viral preparation. Six million human and marmoset PBLs or B-LCLs were challenged with 800,000 (for the PBLs) or 450,000 (for the B-LCLs) reverse transcriptase units (cpm) of VSV-G-pseudotyped HIV-1 GFP reporter viruses in a total volume of 500 μl. As a control, cells were transduced in parallel with viruses lacking the VSV-G envelope glycoprotein. Two hours after transduction, the cells were washed 3 times with phosphate buffered saline (PBS) to eliminate excess virus and resuspended in 3 ml of medium. At different times post-infection, an aliquot containing ~10^6^ cells was collected to isolate the total DNA. The formation of early and late reverse transcription products and 2-LTR circles was measured by real-time qPCR, as previously described^22^,^31^.

### Ethical statement

Human fresh whole blood samples used in this study were obtained from healthy anonymous volunteers through Research Blood Components, LLC (Boston, MA, USA) or the Transfusion Center of the Community of Madrid (Spain). Research Blood Components follows American Association of Blood Banks guidelines for drawing donors. The Transfusion Center of the Community of Madrid follows the Spanish legislation on blood banks to collect samples from donors. In all cases, an Institutional Review Board approved informed consent for drawing the blood samples was obtained from each donor according to local regulations. Confidentiality and lack of donor identification was assured. The usage of human blood samples for this study from these sources was approved by the Dana-Farber Cancer Institute Office for Human Research Studies and the joint Ethics Committee for Research at Centro de Biología Molecular “Severo Ochoa” and Centro Nacional de Biotecnología, respectively.

Blood samples from common marmosets and rhesus macaques were used in this work to study the blocks to HIV-1 replication present in these monkeys. Animals were housed at the New England Primate Research Center, the German Primate Center or the Netherland Biomedical Primate Research Center.

Animals at the New England Primate Research Center were cared for according to the standards of the Association for Assessment and Accreditation of Laboratory Animal Care and the Harvard Medical School Animal Care and Use Committee. Blood collection and procedures on animals at the New England Primate Research Center were approved by the Harvard Medical Area (HMA) Standing Committee on Animals (protocol number 04789) and were in compliance with the Animal Welfare Act and the Public Health Service Policy on Humane Care and Use of Laboratory Animals. The HMA Standing Committee on Animals has an approved Animal Welfare Assurance on file (A3431-01) with the Office for Laboratory Animal Welfare.

Care of and all procedures on the animals performed at the German Primate Center and the Netherlands Biomedical Primate Research Center (BPRC) were done according to local legislation, the European Union Directive 2010/63/EU on the protection of animals used for scientific purposes and the EU recommendations 2007/526/EG for the accommodation and care of animals used for experimental and other scientific purposes. The German Primate Center is registered and authorized by the local and regional veterinary government authorities (reference number 32.22/VO Stadt Göttingen; 392001/7 Stadt Göttingen). Consultation with the Institutional Animal Care and Use Committee (IACUC) is documented under Ethics no. 2–16. Blood sampling of common marmosets at the BPRC was approved by the independent Animal Ethics Committee of the BPRC (Approval #730). The BPRC is AAALAC accredited.

For this study, only non-surgical collection of blood was needed. No other procedures were carried out on the animals for this study. For the phlebotomy, animals were sedated with ketamine HCl (10–50 mg/kg, IM) or with telazol (4–10 mg/kg, IM) to reduce pain and discomfort. The phlebotomy site was prepped with alcohol. Blood samples were obtained from a peripheral vein. The amount of blood collected from any animal as a single sample in a 2-week period did not exceed 10% of the circulating blood volume.

The common marmosets utilized in this study were socially housed. The rhesus macaques were socially housed unless they were being conditioned for other studies or were scheduled for return to the breeding colony. Compensatory enrichment was provided to animals that were not socially housed.

### Statistical analyses

Each experiment was repeated at least twice. The statistical analyses were carried out, when appropriate, with the Excel program. The significance levels (alpha) were set at 0.05. The p values were calculated with a two-tailed t-test. When the equal variance test performed with SigmaPlot failed, the p values were calculated with the unequal variance two-tailed t-test option provided in the Excel program. A Shapiro-Wilk test was performed in all cases to confirm the normality of the data using the SigmaPlot program.

## Additional Information

**How to cite this article**: Pacheco, B. *et al*. Characterization of two distinct early post-entry blocks to HIV-1 in common marmoset lymphocytes. *Sci. Rep.* **6**, 37489; doi: 10.1038/srep37489 (2016).

**Publisher's note:** Springer Nature remains neutral with regard to jurisdictional claims in published maps and institutional affiliations.

## Supplementary Material

Supplementary Figure
