# Supplementary material for: Characterization of two distinct early post-entry blocks to HIV-1 in common marmoset lymphocytes
Source: Sci Rep. 2016 Nov 23;6:37489. doi: 10.1038/srep37489 (PMC5120322; doi:10.1038/srep37489)
Supplement: Supplementary Figure [file srep37489-s1.pdf]

Supplementary Information

Characterization of two distinct early post-entry blocks to HIV-1 in common marmoset lymphocytes

Beatriz Pacheco[1,2], Luis Menéndez-Arias[1] and Joseph Sodroski[2,3]

[1]Centro de Biología Molecular Severo Ochoa, Consejo Superior de Investigaciones Científicas and Universidad Autónoma de Madrid, Madrid, Spain
[2]Department of Cancer Immunology and Virology, Dana-Farber Cancer Institute and Department of Microbiology and Immunobiology, Harvard Medical School, Boston, MA, USA
[3]Department of Immunology and Infectious Diseases, Harvard School of Public Health, Boston, MA, USA

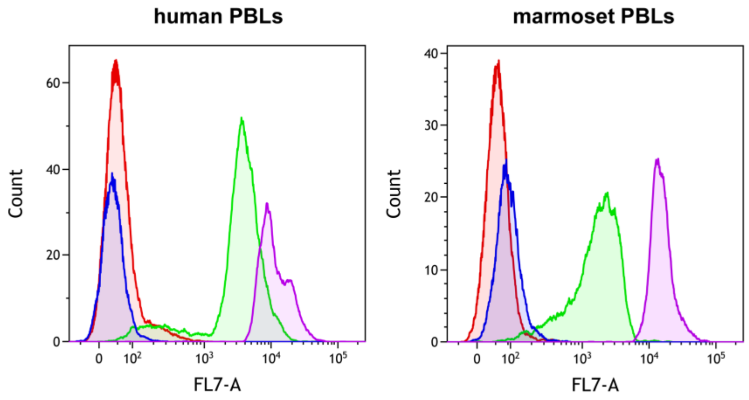

	MFI	
	day 0	day 7
hu PBLs NC	41	67
hu PBLs +CellTrace	12,526	4,047

	MFI	
	day 0	day 7
mar PBLs NC	97	61
mar PBLs +CellTrace	16,061	1,929

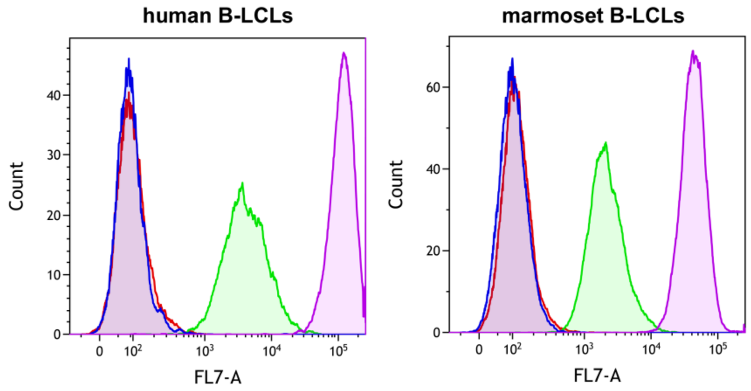

	MFI	
	day 0	day 7
hu B-LCLs NC	106	108
hu B-LCLs +CellTrace	122,179	5,205

	MFI	
	day 0	day 7
mar B-LCLs NC	137	126
mar B-LCLs +CellTrace	45,869	2,520

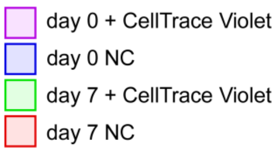

Supplementary Fig S1. Proliferation of PBLs and B-LCLs. Human or marmoset PBLs or B-LCLs were labeled with 1 µM CellTrace Violet dye in PBS for 20 minutes according to manufacturer instructions. The labeled (+CellTrace Violet) and unlabeled (NC) cells were grown for 7 days. The fluorescence of the cells was analyzed by flow cytometry in a FACS Canto A flow cytometer.
